# Poly[diaqua­(μ-oxalato)(μ-2-oxido­pyridinium-3-carboxyl­ato)praseo­dymium(III)]

**DOI:** 10.1107/S160053680900542X

**Published:** 2009-02-21

**Authors:** Yong-Jun Xu, Xiao-Xi Yang, Hong-Bin Zhao

**Affiliations:** a College of Chemistry and Environmental Engineering, Dongguan University of Technology, Dongguan 523808, Guangdong, People’s Republic of China

## Abstract

In the title complex, [Pr(C_6_H_4_NO_3_)(C_2_O_4_)(H_2_O)_2_]_*n*_, each Pr^III^ ion is coordinated by eight O atoms from two 2-oxynicotinate ligands, two oxalate ligands and two water mol­ecules, displaying a distorted bicapped square-anti­prismatic geometry. The carboxyl­ate groups link adjacent praseodymium metal centres, forming layers parallel to the *bc* plane. The crystal packing is stabilized by inter­molecular O—H⋯O and N—H⋯O hydrogen bonds.

## Related literature

For a general background on the mol­ecular self-assembly of supra­molecular architectures, see: Mou *et al.* (2008 ▶); Moulton & Zaworotko (2001 ▶); Zeng *et al.* (2007 ▶).
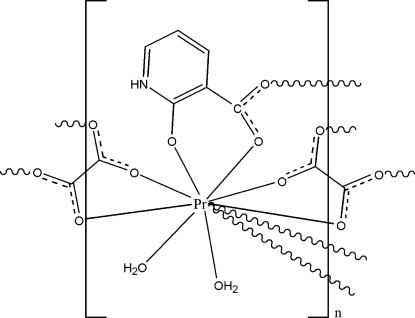


## Experimental

### 

#### Crystal data

[Pr(C_6_H_4_NO_3_)(C_2_O_4_)(H_2_O)_2_]
*M* *_r_* = 403.06
Triclinic, 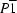

*a* = 7.5820 (19) Å
*b* = 8.643 (2) Å
*c* = 9.375 (4) Å
α = 108.992 (4)°
β = 103.925 (4)°
γ = 102.043 (3)°
*V* = 535.6 (3) Å^3^
*Z* = 2
Mo *K*α radiation
μ = 4.60 mm^−1^
*T* = 296 K
0.19 × 0.17 × 0.16 mm

#### Data collection

Bruker APEXII area-detector diffractometer
Absorption correction: multi-scan (*SADABS*; Sheldrick, 1996 ▶) *T*_min_ = 0.435, *T*_max_ = 0.485
2751 measured reflections
1888 independent reflections
1753 reflections with *I* > 2σ(*I*)
*R*_int_ = 0.024

#### Refinement

*R*[*F* ^2^ > 2σ(*F* ^2^)] = 0.030
*wR*(*F* ^2^) = 0.080
*S* = 1.10
1888 reflections
187 parameters
8 restraints
H atoms treated by a mixture of independent and constrained refinement
Δρ_max_ = 1.06 e Å^−3^
Δρ_min_ = −1.66 e Å^−3^

### 

Data collection: *APEX2* (Bruker, 2004 ▶); cell refinement: *APEX2*; data reduction: *SAINT* (Bruker, 2004 ▶); program(s) used to solve structure: *SHELXS97* (Sheldrick, 2008 ▶); program(s) used to refine structure: *SHELXL97* (Sheldrick, 2008 ▶); molecular graphics: *SHELXTL* (Sheldrick, 2008 ▶); software used to prepare material for publication: *SHELXL97*.

## Supplementary Material

Crystal structure: contains datablocks I, global. DOI: 10.1107/S160053680900542X/rz2293sup1.cif

Structure factors: contains datablocks I. DOI: 10.1107/S160053680900542X/rz2293Isup2.hkl

Additional supplementary materials:  crystallographic information; 3D view; checkCIF report

## Figures and Tables

**Table 1:** Hydrogen-bond geometry (Å, °)

*D*—H⋯*A*	*D*—H	H⋯*A*	*D*⋯*A*	*D*—H⋯*A*
O2*W*—H4*W*⋯O3	0.83 (3)	2.56 (2)	3.306 (6)	149 (4)
O2*W*—H3*W*⋯O7^i^	0.84 (5)	1.95 (6)	2.764 (6)	164 (7)
O2*W*—H4*W*⋯O2*W*^ii^	0.83 (3)	2.37 (2)	2.820 (8)	114 (2)
O1*W*—H2*W*⋯O2^ii^	0.85 (6)	2.49 (4)	3.280 (7)	157 (8)
O1*W*—H1*W*⋯O3	0.84 (5)	2.34 (6)	2.760 (6)	111 (5)
O1*W*—H1*W*⋯O6^iii^	0.84 (5)	2.24 (3)	3.002 (6)	151 (6)
N1—H1⋯O4^iii^	0.86 (6)	2.12 (4)	2.878 (7)	146 (6)

Symmetry codes: (i) 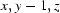; (ii) 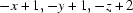; (iii) 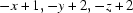.

**Fig. 1.**
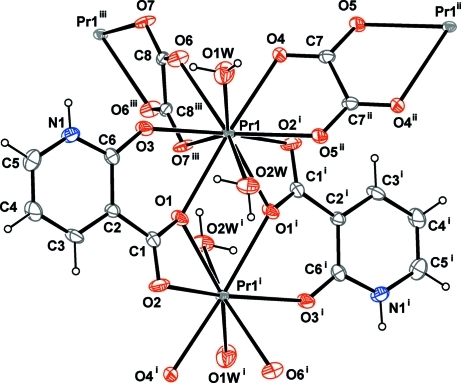
The molecular structure of the title compound showing the atom-numbering scheme. Displacement ellipsoids are drawn at the 50% probability level. Symmetry codes: (i) -x, 1 - y, 2 - z; (ii) -x, 1 - y, 1 - z; (iii) -x, 2 - y, 2 - z.

**Fig. 2.**
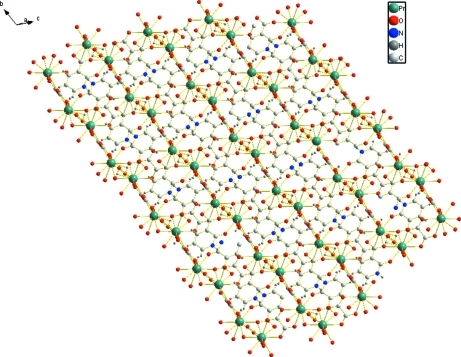
Crystal structure of the title compound viewed approximately along the a axis.

## supplementary crystallographic information

### Comment

Molecular self-assembly of supramolecular architectures has received much attention during recent decades (Zeng *et al.*, 2007; Moulton & Zaworotko, 2001; Mou *et al.*, 2008). The structures and properties of such systems depend on the coordination and geometric preferences of both the central metal ions and the bridging building blocks, as well as the influence of weaker non-covalent interactions, such as hydrogen bonds and π-π stacking interactions. Recently, we obtained the title coordination polymer, which was synthesized under hydrothermal conditions.

As illustrated in Fig. 1, in the structure of the title compound each Pr^III^ centre is in a distorted bicapped square antiprismatic geometry, defined by eight oxygen atoms from two 2-oxynicotinate ligands, two oxalate ligands, and two water molecules The Pr^III^ ions are linked by 2-oxynicotinate ligands and oxalate ligands to form layers parallel to the *bc* plane (Fig. 2), with separations between adjacent Pr^III^ metal centres of 4.410 (4), 6.505 (5) and 6.551 (3) Å. Intermolecular O—H···O and N—H···O hydrogen bonding interactions (Table 1) involving the 2-oxynicotinate ligands, the oxalate ligands and the water molecules assemble neighboring layers to form a three-dimensional supramolecular network motif.

### Experimental

A mixture of Pr_2_O_3_ (0.330 g; 1.0 mmol), 2-oxynicotinic acid (0.127 g; 1 mmol), oxalic acid(0.09 g; 1 mmol), water (10 ml) in the presence of HNO_3_ (0.024 g; 0.385 mmol) was stirred vigorously for 20 min and then sealed in a Teflon-lined stainless-steel autoclave (20 ml, capacity). The autoclave was heated and maintained at 443 K for 3 days. After cooling to room temperature at 5 K h^-1^, colourless block crystals of the title compound were obtained.

### Refinement

Water H atoms were located in difference Fourier maps and were refined with distance restraints of O–H = 0.84 Å, H···H = 1.35 Å, and with *U*_iso_(H) = 1.5 *U*_eq_(O). The separation between symmetry related H4W atoms at (*x*, *y*, *z*) and (1 - *x*, 1 - *y*, 2 - *z*) was restrained to be 2.2 Å. Carbon-bound H atoms were placed at calculated positions and were treated as riding on the parent C atoms with C—H = 0.93 Å, and with *U*_iso_(H) = 1.2 *U*_eq_(C). The H atom bound to the N1 nitrogen atom was refined with a distance restraints of N–H = 0.86 Å and with *U*_iso_(H) = 1.2 *U*_eq_(N).

### Crystal data

**Table:**

[Pr(C_6_H_4_NO_3_)(C_2_O_4_)(H_2_O)_2_]	*Z* = 2
*M**_r_* = 403.06	*F*(000) = 388
Triclinic, *P*1	*D*_x_ = 2.499 Mg m^−^^3^
Hall symbol: -P 1	Mo *K*α radiation, λ = 0.71073 Å
*a* = 7.5820 (19) Å	Cell parameters from 2183 reflections
*b* = 8.643 (2) Å	θ = 2.6–28.0°
*c* = 9.375 (4) Å	µ = 4.60 mm^−^^1^
α = 108.992 (4)°	*T* = 296 K
β = 103.925 (4)°	Block, colourless
γ = 102.043 (3)°	0.19 × 0.17 × 0.16 mm
*V* = 535.6 (3) Å^3^	

### Data collection

**Table:**

Bruker APEXII area-detector diffractometer	1888 independent reflections
Radiation source: fine-focus sealed tube	1753 reflections with *I* > 2σ(*I*)
graphite	*R*_int_ = 0.024
φ and ω scans	θ_max_ = 25.2°, θ_min_ = 2.4°
Absorption correction: multi-scan (*SADABS*; Sheldrick, 1996)	*h* = −9→8
*T*_min_ = 0.435, *T*_max_ = 0.485	*k* = −6→10
2751 measured reflections	*l* = −11→10

### Refinement

**Table:**

Refinement on *F*^2^	Primary atom site location: structure-invariant direct methods
Least-squares matrix: full	Secondary atom site location: difference Fourier map
*R*[*F*^2^ > 2σ(*F*^2^)] = 0.030	Hydrogen site location: inferred from neighbouring sites
*wR*(*F*^2^) = 0.080	H atoms treated by a mixture of independent and constrained refinement
*S* = 1.10	*w* = 1/[σ^2^(*F*_o_^2^) + (0.0483*P*)^2^ + 0.01*P*] where *P* = (*F*_o_^2^ + 2*F*_c_^2^)/3
1888 reflections	(Δ/σ)_max_ = 0.001
187 parameters	Δρ_max_ = 1.06 e Å^−^^3^
8 restraints	Δρ_min_ = −1.66 e Å^−^^3^

### Special details

**Table:**

Geometry. All e.s.d.'s (except the e.s.d. in the dihedral angle between two l.s. planes) are estimated using the full covariance matrix. The cell e.s.d.'s are taken into account individually in the estimation of e.s.d.'s in distances, angles and torsion angles; correlations between e.s.d.'s in cell parameters are only used when they are defined by crystal symmetry. An approximate (isotropic) treatment of cell e.s.d.'s is used for estimating e.s.d.'s involving l.s. planes.
Refinement. Refinement of *F*^2^ against ALL reflections. The weighted *R*-factor *wR* and goodness of fit *S* are based on *F*^2^, conventional *R*-factors *R* are based on *F*, with *F* set to zero for negative *F*^2^. The threshold expression of *F*^2^ > σ(*F*^2^) is used only for calculating *R*-factors(gt) *etc*. and is not relevant to the choice of reflections for refinement. *R*-factors based on *F*^2^ are statistically about twice as large as those based on *F*, and *R*- factors based on ALL data will be even larger.

### Fractional atomic coordinates and isotropic or equivalent isotropic displacement parameters (Å^2^)

**Table:**

	*x*	*y*	*z*	*U*_iso_*/*U*_eq_	
Pr1	0.13688 (4)	0.65736 (3)	0.88899 (3)	0.01199 (13)	
O1	0.1360 (5)	0.5899 (6)	1.1378 (4)	0.0209 (9)	
O2	0.2431 (6)	0.4573 (6)	1.2882 (5)	0.0292 (10)	
O3	0.4163 (6)	0.8435 (6)	1.1280 (4)	0.0255 (10)	
O4	0.0792 (6)	0.7141 (5)	0.6359 (4)	0.0210 (9)	
O5	−0.0653 (6)	0.5985 (5)	0.3701 (5)	0.0240 (9)	
O6	0.1917 (5)	0.9740 (5)	0.9339 (5)	0.0211 (9)	
O7	0.0683 (6)	1.1925 (5)	0.9755 (5)	0.0221 (9)	
N1	0.6714 (7)	0.9795 (7)	1.3550 (6)	0.0213 (11)	
H1	0.709 (9)	1.056 (6)	1.320 (7)	0.026*	
C1	0.2690 (8)	0.5814 (8)	1.2460 (6)	0.0184 (12)	
C2	0.4526 (7)	0.7259 (7)	1.3291 (6)	0.0155 (11)	
C3	0.5671 (8)	0.7451 (8)	1.4747 (7)	0.0226 (13)	
H3	0.5331	0.6633	1.5160	0.027*	
C4	0.7339 (8)	0.8845 (9)	1.5629 (7)	0.0268 (14)	
H4	0.8106	0.8963	1.6620	0.032*	
C5	0.7816 (8)	1.0019 (8)	1.5011 (7)	0.0260 (14)	
H5	0.8900	1.0978	1.5591	0.031*	
C6	0.5042 (8)	0.8480 (8)	1.2612 (6)	0.0176 (12)	
C7	0.0043 (7)	0.5910 (7)	0.5025 (6)	0.0169 (12)	
C8	0.0756 (7)	1.0482 (7)	0.9731 (6)	0.0132 (11)	
O1W	0.4579 (6)	0.7364 (7)	0.8300 (6)	0.0344 (11)	
H1W	0.526 (8)	0.825 (6)	0.912 (6)	0.052*	
H2W	0.532 (7)	0.703 (9)	0.781 (7)	0.052*	
O2W	0.3042 (6)	0.4283 (6)	0.9073 (5)	0.0256 (9)	
H3W	0.253 (9)	0.355 (5)	0.938 (6)	0.038*	
H4W	0.362 (3)	0.520 (3)	0.987 (4)	0.038*	

### Atomic displacement parameters (Å^2^)

**Table:**

	*U*^11^	*U*^22^	*U*^33^	*U*^12^	*U*^13^	*U*^23^
Pr1	0.01204 (18)	0.0123 (2)	0.01350 (19)	0.00367 (13)	0.00302 (12)	0.00841 (14)
O1	0.0147 (19)	0.030 (3)	0.0152 (19)	0.0010 (18)	−0.0001 (16)	0.0128 (18)
O2	0.030 (2)	0.023 (3)	0.031 (2)	−0.003 (2)	0.0026 (19)	0.019 (2)
O3	0.0211 (19)	0.029 (3)	0.019 (2)	−0.0046 (18)	−0.0042 (16)	0.0153 (19)
O4	0.031 (2)	0.015 (2)	0.0137 (19)	0.0041 (18)	0.0044 (17)	0.0060 (17)
O5	0.040 (2)	0.016 (2)	0.016 (2)	0.0109 (19)	0.0039 (18)	0.0103 (18)
O6	0.0183 (19)	0.019 (2)	0.035 (2)	0.0104 (18)	0.0150 (17)	0.0138 (19)
O7	0.027 (2)	0.013 (2)	0.036 (2)	0.0079 (18)	0.0173 (18)	0.0158 (19)
N1	0.019 (2)	0.019 (3)	0.025 (3)	0.002 (2)	0.003 (2)	0.012 (2)
C1	0.023 (3)	0.019 (3)	0.013 (3)	0.006 (3)	0.005 (2)	0.006 (2)
C2	0.015 (2)	0.018 (3)	0.015 (3)	0.004 (2)	0.006 (2)	0.008 (2)
C3	0.026 (3)	0.028 (4)	0.018 (3)	0.009 (3)	0.007 (2)	0.016 (3)
C4	0.020 (3)	0.035 (4)	0.019 (3)	0.005 (3)	−0.003 (2)	0.012 (3)
C5	0.015 (3)	0.025 (4)	0.029 (3)	0.003 (3)	0.001 (2)	0.006 (3)
C6	0.019 (3)	0.021 (3)	0.015 (3)	0.008 (2)	0.008 (2)	0.006 (2)
C7	0.017 (3)	0.017 (3)	0.019 (3)	0.005 (2)	0.005 (2)	0.011 (2)
C8	0.014 (2)	0.011 (3)	0.013 (3)	0.003 (2)	0.000 (2)	0.005 (2)
O1W	0.025 (2)	0.035 (3)	0.034 (3)	0.000 (2)	0.0112 (19)	0.007 (2)
O2W	0.025 (2)	0.030 (3)	0.033 (2)	0.015 (2)	0.0120 (18)	0.021 (2)

### Geometric parameters (Å, °)

**Table:**

Pr1—O3	2.458 (4)	O7—C8	1.253 (7)
Pr1—O4	2.532 (4)	O7—Pr1^i^	2.537 (4)
Pr1—O7^i^	2.537 (4)	N1—C5	1.350 (8)
Pr1—O5^ii^	2.540 (4)	N1—C6	1.372 (8)
Pr1—O1^iii^	2.543 (4)	N1—H1	0.86 (6)
Pr1—O6	2.555 (4)	C1—C2	1.491 (8)
Pr1—O1	2.583 (4)	C1—Pr1^iii^	3.018 (6)
Pr1—O2W	2.593 (4)	C2—C3	1.367 (8)
Pr1—O1W	2.626 (4)	C2—C6	1.434 (8)
Pr1—O2^iii^	2.734 (4)	C3—C4	1.396 (8)
Pr1—C1^iii^	3.018 (6)	C3—H3	0.9300
Pr1—H4W	2.44 (4)	C4—C5	1.352 (9)
O1—C1	1.280 (7)	C4—H4	0.9300
O1—Pr1^iii^	2.543 (4)	C5—H5	0.9300
O2—C1	1.253 (7)	C7—C7^ii^	1.546 (11)
O2—Pr1^iii^	2.734 (4)	C8—C8^i^	1.555 (10)
O3—C6	1.251 (7)	O1W—H1W	0.84 (5)
O4—C7	1.248 (7)	O1W—H2W	0.84 (5)
O5—C7	1.255 (6)	O2W—H3W	0.84 (5)
O5—Pr1^ii^	2.540 (4)	O2W—H4W	0.83 (3)
O6—C8	1.249 (6)		
			
O3—Pr1—O4	120.86 (13)	O4—Pr1—H4W	127.8 (11)
O3—Pr1—O7^i^	88.47 (14)	O7^i^—Pr1—H4W	129.5 (10)
O4—Pr1—O7^i^	102.37 (13)	O5^ii^—Pr1—H4W	81.2 (5)
O3—Pr1—O5^ii^	137.09 (15)	O1^iii^—Pr1—H4W	88.8 (9)
O4—Pr1—O5^ii^	63.36 (13)	O6—Pr1—H4W	129.1 (6)
O7^i^—Pr1—O5^ii^	134.11 (14)	O1—Pr1—H4W	60.0 (11)
O3—Pr1—O1^iii^	127.58 (12)	O2W—Pr1—H4W	18.7 (8)
O4—Pr1—O1^iii^	111.40 (13)	O1W—Pr1—H4W	67.4 (11)
O7^i^—Pr1—O1^iii^	76.36 (13)	O2^iii^—Pr1—H4W	134.2 (6)
O5^ii^—Pr1—O1^iii^	70.57 (14)	C1^iii^—Pr1—H4W	111.2 (8)
O3—Pr1—O6	69.27 (14)	C1—O1—Pr1^iii^	98.8 (3)
O4—Pr1—O6	65.37 (13)	C1—O1—Pr1	131.9 (3)
O7^i^—Pr1—O6	63.08 (12)	Pr1^iii^—O1—Pr1	118.71 (14)
O5^ii^—Pr1—O6	128.42 (12)	C1—O2—Pr1^iii^	90.5 (3)
O1^iii^—Pr1—O6	136.28 (12)	C6—O3—Pr1	140.0 (4)
O3—Pr1—O1	66.31 (12)	C7—O4—Pr1	119.9 (3)
O4—Pr1—O1	170.59 (12)	C7—O5—Pr1^ii^	120.3 (4)
O7^i^—Pr1—O1	70.75 (13)	C8—O6—Pr1	120.7 (3)
O5^ii^—Pr1—O1	116.45 (13)	C8—O7—Pr1^i^	121.3 (3)
O1^iii^—Pr1—O1	61.29 (14)	C5—N1—C6	125.6 (5)
O6—Pr1—O1	115.03 (13)	C5—N1—H1	116 (5)
O3—Pr1—O2W	81.72 (14)	C6—N1—H1	118 (4)
O4—Pr1—O2W	117.75 (13)	O2—C1—O1	120.7 (5)
O7^i^—Pr1—O2W	137.99 (13)	O2—C1—C2	119.7 (5)
O5^ii^—Pr1—O2W	63.32 (13)	O1—C1—C2	119.5 (5)
O1^iii^—Pr1—O2W	77.80 (13)	O2—C1—Pr1^iii^	65.0 (3)
O6—Pr1—O2W	144.55 (13)	O1—C1—Pr1^iii^	56.4 (3)
O1—Pr1—O2W	67.88 (13)	C2—C1—Pr1^iii^	168.4 (4)
O3—Pr1—O1W	65.67 (14)	C3—C2—C6	120.3 (5)
O4—Pr1—O1W	69.36 (14)	C3—C2—C1	119.0 (5)
O7^i^—Pr1—O1W	139.13 (15)	C6—C2—C1	120.6 (5)
O5^ii^—Pr1—O1W	79.60 (14)	C2—C3—C4	121.5 (6)
O1^iii^—Pr1—O1W	144.47 (15)	C2—C3—H3	119.2
O6—Pr1—O1W	77.88 (14)	C4—C3—H3	119.2
O1—Pr1—O1W	120.06 (14)	C5—C4—C3	118.5 (5)
O2W—Pr1—O1W	71.70 (15)	C5—C4—H4	120.7
O3—Pr1—O2^iii^	153.63 (15)	C3—C4—H4	120.7
O4—Pr1—O2^iii^	67.95 (13)	N1—C5—C4	119.8 (6)
O7^i^—Pr1—O2^iii^	65.16 (14)	N1—C5—H5	120.1
O5^ii^—Pr1—O2^iii^	69.19 (14)	C4—C5—H5	120.1
O1^iii^—Pr1—O2^iii^	49.16 (12)	O3—C6—N1	118.7 (5)
O6—Pr1—O2^iii^	96.62 (13)	O3—C6—C2	127.0 (5)
O1—Pr1—O2^iii^	102.91 (12)	N1—C6—C2	114.3 (5)
O2W—Pr1—O2^iii^	117.69 (14)	O4—C7—O5	127.0 (5)
O1W—Pr1—O2^iii^	135.00 (14)	O4—C7—C7^ii^	117.4 (6)
O3—Pr1—C1^iii^	147.53 (14)	O5—C7—C7^ii^	115.7 (6)
O4—Pr1—C1^iii^	88.65 (14)	O6—C8—O7	127.4 (5)
O7^i^—Pr1—C1^iii^	70.79 (14)	O6—C8—C8^i^	116.4 (6)
O5^ii^—Pr1—C1^iii^	65.76 (14)	O7—C8—C8^i^	116.2 (6)
O1^iii^—Pr1—C1^iii^	24.77 (14)	Pr1—O1W—H1W	103 (4)
O6—Pr1—C1^iii^	118.47 (14)	Pr1—O1W—H2W	148 (5)
O1—Pr1—C1^iii^	83.03 (13)	H1W—O1W—H2W	107 (6)
O2W—Pr1—C1^iii^	96.95 (15)	Pr1—O2W—H3W	116 (5)
O1W—Pr1—C1^iii^	144.76 (15)	Pr1—O2W—H4W	70 (3)
O2^iii^—Pr1—C1^iii^	24.54 (14)	H3W—O2W—H4W	108 (4)
O3—Pr1—H4W	63.2 (7)		

Symmetry codes: (i) −*x*, −*y*+2, −*z*+2; (ii) −*x*, −*y*+1, −*z*+1; (iii) −*x*, −*y*+1, −*z*+2.

### Hydrogen-bond geometry (Å, °)

**Table:**

*D*—H···*A*	*D*—H	H···*A*	*D*···*A*	*D*—H···*A*
O2W—H4W···O3	0.83 (3)	2.56 (2)	3.306 (6)	149 (4)
O2W—H3W···O7^iv^	0.84 (5)	1.95 (6)	2.764 (6)	164 (7)
O2W—H4W···O2W^v^	0.83 (3)	2.37 (2)	2.820 (8)	114 (2)
O1W—H2W···O2^v^	0.85 (6)	2.49 (4)	3.280 (7)	157 (8)
O1W—H1W···O3	0.84 (5)	2.34 (6)	2.760 (6)	111 (5)
O1W—H1W···O6^vi^	0.84 (5)	2.24 (3)	3.002 (6)	151 (6)
N1—H1···O4^vi^	0.86 (6)	2.12 (4)	2.878 (7)	146 (6)

Symmetry codes: (iv) *x*, *y*−1, *z*; (v) −*x*+1, −*y*+1, −*z*+2; (vi) −*x*+1, −*y*+2, −*z*+2.
